# Ureteral anastomosis with a polyimide stent in rat kidney transplantation

**DOI:** 10.1080/0886022X.2020.1726386

**Published:** 2020-02-13

**Authors:** Tong Wang, Zhou Yu, Chen Chen, Yajuan Song, Xianhui Zeng, Yingjun Su, Chenggang Yi

**Affiliations:** a Department of Plastic Surgery; Xijing Hospital, Air Force Medical University, Xi’an, China; b Department of Burn and Plastic Surgery, Hainan Branch of PLA General Hospital, Sanya, China

**Keywords:** Kidney transplant, ureteral anastomosis, stent, polyimide tube

## Abstract

**Background:**

Complications associated with ureteral anastomosis in kidney transplantation are highly prevalent, despite the development of various types of stents. The current stent materials and placement methods have several limitations. This study attempts to provide an alternative by investigating ureteral anastomosis with a polyimide stent and a modified placement method in a rat model of kidney transplantation.

**Methods:**

Sprague–Dawley rats were randomly divided into Group I: sham operation, Group II: autologous ureteral anastomosis, and Group III: isogenic kidney transplantation with ureteral anastomosis. For the anastomosis, a polyimide stent with a previously placed 11-0 silk was inserted into the ureter. The stent and ureter were fixed with 11-0 silk sutures. The kidney weight and serum creatinine were recorded. The ureteral and renal sections were taken for histological analysis.

**Results:**

None of the stents had migrated. Urethral patency was achieved. Further, there were no evident histological changes in the anastomosed ureters. The serum creatinine level in group III was significantly higher than the other two groups, but there was no significant difference in kidney weight among the groups at postoperative week 12. Finally, the histological structure of kidneys in groups II and III only showed minor changes.

**Conclusions:**

The current anastomosis method with polyimide stent causes minimal damage to the ureteral walls and minimizes the possibility of stent migration. Therefore, this method of ureteral anastomosis with the polyimide stent should be explored for its potential benefits in more animal kidney transplantation models, thus providing an alternative for the clinical setting.

## Introduction

Kidney transplantation involves ureteric reconstruction procedures such as bladder insertion, bladder patch, and end-to-end anastomosis without and with stent placement [1–11]. These reconstruction procedures are associated with several urinary complications: for example, ureteral anastomosis using bladder insertion and bladder patch can result in ureteric necrosis and fistula, and end-to-end anastomosis without stent placement can cause ureteric kinking, fistula, and hemorrhage. Such complications occur in 10–20% of kidney transplantations [1], and they could eventually lead to kidney loss or even recipient death. Thus, ureteral reconstruction remains a challenge in renal transplantation.

Ureteral stents of various materials have been used in ureteral anastomosis [12]. However, these stents have several limitations that prevent their widespread deployment in kidney transplantation. One of the general limitations is the relative stiffness of these materials, which when combined with the upward or downward sliding motion of the stent, can cause damage to the thin ureteral wall. Further, obstructions were found in 77.8% of ureters that were anastomosed with a polyethylene tube stent [7], and blood clots and perforation of the ureteric wall were observed in ureters that were reconstructed with a silastic catheter [4]. Another complication is pyelectasis, which occurred in 80% of ureter reconstructions with nylon stents [4]. The long-term implantation of stents is also associated with stone formation and hydronephrosis [3]. Besides, the PCL/PLGA stent was also placed into the ureter. Although it showed excellent biocompatibility, a few stent-associated symptoms were still observed [13, 14]. Therefore, there is an urgent need to develop a stent that can overcome these limitations and prevent or reduce the associated complications.

Polyimide is considered to be an ideal polymer material for stents on account of its high thermal stability, excellent chemical and physical properties, good adhesion properties, and good biocompatibility. Polyimide is currently used for the production of medical devices and implants, such as microelectrodes, chemical sensors, microchannels, and blood pressure sensors. In addition, a stent made from polyimide tubing has been successfully used for vascular anastomosis in vascularized skin transplantation performed in a mouse model [15], with no evidence of damage or thrombus formation. The ureter and blood vessels share similarities in terms of their composition and liquid transport function. Therefore, a polyimide stent may also be suitable for ureteral anastomosis during renal transplantation.

Rat kidney transplantation is an important model for the study of immunological rejection, immunologic tolerance, and transplantation-associated diseases. In this model, ureteral anastomosis is necessary. And the utilization of polyimide stent for ureteral anastomosis has not been reported. Therefore, in the present study, we have used this model to examine the feasibility of a new stent technique with a polyimide tube for ureteral anastomosis in rats and monitored the survival of the transplanted kidney and renal function after isograft kidney transplantation.

## Methods

### Animals and experimental design

Adult male Sprague–Dawley rats, weighing 250 – 300 g, were used as the animal models in this study. They were purchased from the Experimental Animal Center of the Air Force Medical University. All animals were kept in climate-controlled animal rooms (temperature, 23 ± 2 °C; humidity, 55 ± 10%) with 12-h light/dark cycles. They were not fed anything for 12 h before the experiment, but they had free access to water. All the animals were randomly divided into three groups: Group I: sham operation (*n* = 21), Group II: autologous ureter anastomosis (*n* = 21), and Group III: isogenic kidney transplantation with ureteral anastomosis (*n* = 21). All the rats were anesthetized with pentobarbital sodium (50 mg/kg body weight, IP) and placed on the operating table in the supine position, with their body temperature maintained at 37 °C on a heating pad. The procedures were performed under a binocular operating microscope (Leica M651; Leica Microsystems AG, Heerbrugg, Switzerland) by a single operator.

The animal experimental protocol was approved by the Experimental Animal Committee of the Air Force Medical University (No. 20181110).

### Ureter stent preparation

Polyimide tubes (River Tech Medical, Chatanooga, TN) were cut to a 3-mm length (inner diameter, 0.4572 mm; wall thickness, 0.0254 mm). The oblique ends and end surfaces were subjected to passivation.

### Surgical protocol

**Figure 1.**
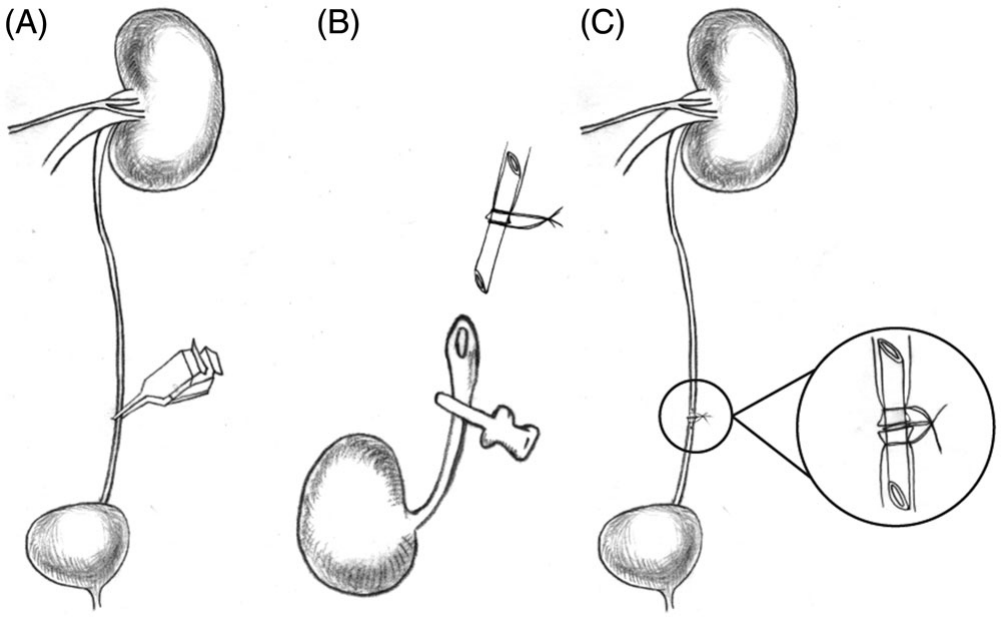
Schematic diagram of the ureteral anastomosis procedure with polyimide stent placement. (A) The left ureter was blocked for 1-h to induce dilatation of the ureter. (B) The stent was placed into the left ureter and tied with a 11-0 silk suture. (C) The three ligatures were tied together.

Group I: The animals in the first group underwent a sham operation. In this procedure, a long abdominal incision was made from the xyphoid to the symphysis pubis. The bowel was retracted to the right and covered with a moist gauze. The left ureter was exposed and dissected from the kidney to the bladder by a sharp dissection and freed from the retroperitoneum. Care was taken to preserve the periureteral connective tissue and fat in order to protect ureteral blood supply. A micro-vascular clip was used to block the ureter at the distal point to induce dilatation of the ureter for 1 h (Figure 1(A)). Then, the bowel was replaced in its original position, and the abdomen was closed in two layers using a 5-0 sterile nonabsorbable suture.

Group II: The animals in this group underwent autologous ureteral anastomosis. The rats were prepared as described for group I. After the 1-h dilation, the left ureter was dissected down the middle into two parts, and the stent, with a previously placed 11-0 silk, was placed into the ureter (Figure 1(B)). Both ends of the ureter were fixed with 11-0 silk suture. After this, the three ligatures were tied together to confirm the ureteral anastomosis (Figure 1(C)). Then, the bowel was replaced in its original position, and the abdomen was closed in two layers using a 5-0 sterile nonabsorbable suture.

Group III (donor animals): Donor rats were prepared as described for group II. Briefly, the left ureter was ligated and cut at a point that was 15 mm distal to the left kidney, after the mobilization of adipose tissue around the left kidney and ureter. The left kidney, abdominal aorta (AO), and inferior vena cava (IVC) were exposed. The vessels of the left adrenal grand and left testis were ligated with an 11-0 silk suture. After mobilization of the IVC and AO, the renal artery and renal vein were dissected and isolated, respectively. The lower and upper levels of the AO were clamped near the left renal pedicle. A 25-gauge needle was inserted into the distal end of the AO, and the left kidney was perfused with approximately 10 mL of ice-cold heparin solution until the kidney was uniformly pale. Then, the renal veins were clamped and cut close to their junctions, and the AO was cut at two places (proximal and distal to the left renal artery). The harvested kidney was immediately transferred to ice-cold isotonic saline.

Group III (recipient animals): The recipient rats were prepared in the same manner as the donor rats. A micro-vascular clip was used to block the ureter at the distal point to dilate the ureter. After mobilization and ligation of the lumbar branches, the AO and IVC were clamped from the left renal artery and vein to the iliac bifurcation. The kidney was placed to the left of the recipient abdomen. The grafted kidney was covered with gauze, and ice chips were placed over the gauze. The graft was continuously instilled with ice-cold physiologic saline solution. Under a 12× binocular operating microscope, arterial and venous reconstruction was performed with end-to-side anastomosis using an 11-0 uninterrupted nylon suture. After revascularization, all the clamps were released to restore blood supply to the donor kidney. The color of the graft turned to an even and bright pink, RA pulsation was obvious, and the RV was engorged. Then, a left nephrectomy was performed. When clear urine was observed passing through the ureterostoma, the ureter was immediately anastomosed with the stent (as described in group II). Finally, the upper and lower poles of the donor kidney were fixed with a 9-0 suture line.

After the procedure, 2 mL of saline was injected into the abdominal cavity of animals in all the groups, and they were separately caged under sterile conditions. Right nephrectomy was performed on postoperative day 7 in groups II and III. Immunosuppressive therapy was administered in the graft recipients (group III) with cyclosporine A (10 mg/kg body weight per day) injected intraperitoneally for 10 days after the operation.

### Evaluation parameters

The time for ureter reconstruction was recorded in groups II and III. The diameters of the ureter before and after dilatation were recorded as well. The number of survivors in each group was recorded on postoperative day 7. At 4, 8, and 12 weeks after the procedure, the kidneys were weighed.

### Ureteral function

To evaluate the possibility of leakage or stenosis, methylene blue was injected *via* the upper ureter into the ureter at 12 weeks after ureteral reconstruction.

### Renal function

Blood samples were drawn through the inferior vena cava at 4, 8, and 12 weeks after the procedure for serum creatinine analysis.

### Histological evaluation

Kidney and ureter samples were obtained and fixed with formalin. Then, the paraffin-embedded blocks were cut into 4-μm-thick sections for hematoxylin and eosin staining. Brightfield images were obtained using a Nikon Eclipse 50i microscope, with adjustments for brightness and contrast with Photoshop CS3 (Adobe Systems, San Jose, CA).

### Statistical analysis

Statistical analysis was performed using Prism, version 6.0 (GraphPad Software Inc., CA). The results were reported as the mean ± standard deviation (mean ± SEM), and the difference between the experimental and control group was determined by ANOVA. *p* values less than .05 were considered to indicate statistical significance.

## Results

### Survival and postoperative complications

**Figure 2.**
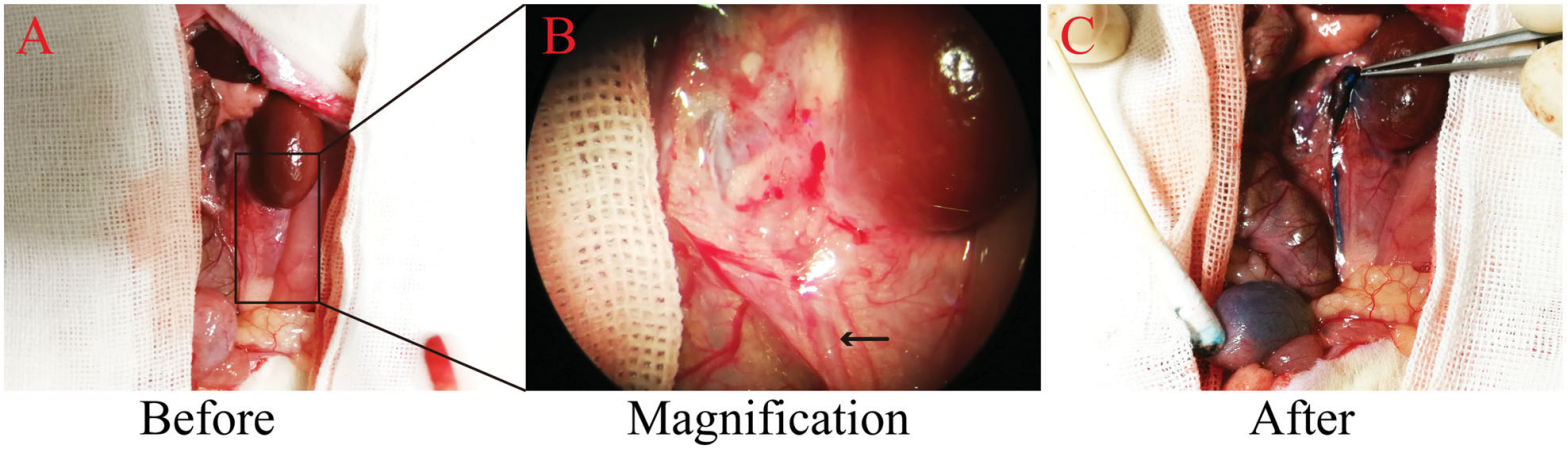
Urethral patency at 12 weeks after anastomosis with stent placement. The patency is demonstrated by the free passage of injected methylene blue solution. (A) Upper ureter before injection of methylene blue. (B) An enlarged view of the area marked out in panel A. The arrow indicates the ureter. (C) Upper ureter after the injection of methylene blue solution.

End-to-end ureter anastomosis was successfully performed using the polyimide stent. At the end point of the experiment, all the rats had tolerated the procedure and survived. The mean total ureter anastomosis time was (2 ± 0.35) min. The diameters of the ureter before and after dilatation were (0.728 ± 0.08) mm and (1.16 ± 0.29) mm, respectively. At 4w, 8w, and 12w after the procedure, the stents were in place, but the reconstruction area was coated with fibrous tissue. The proximal end of the ureter was slightly dilated in group III. Free passage of the injected methylene blue solution through ureter to the bladder was observed (Figure 2). These results are indicative of normal ureteral caliber and urethral patency, without leakage, or stenosis.

**Figure 3.**
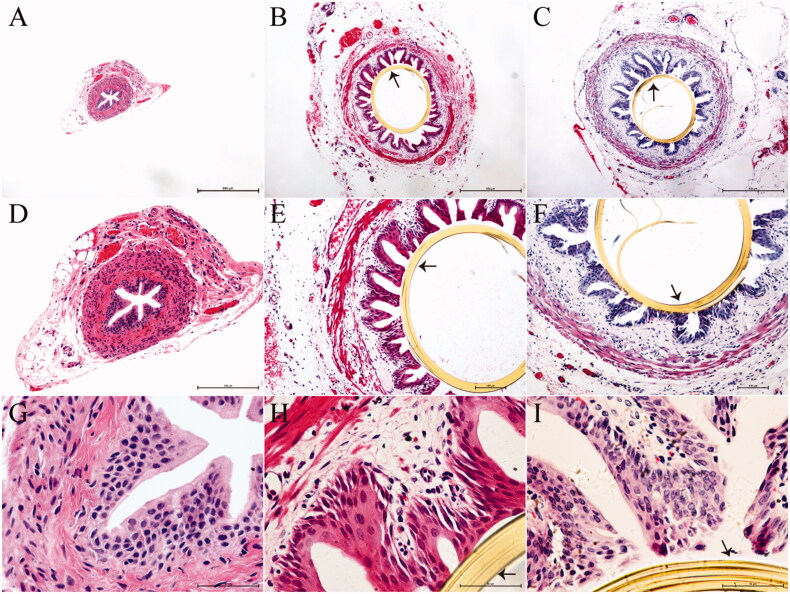
Histopathological evaluation of ureters fixed with the polyimide stent at postoperative week 12. Panels A, B, and C are images acquired at a magnification of 4×, panels D, E, and F represent the same images acquired at a magnification of 10×, and panels G, H, and I represent the same images acquired at a magnification of 40×, (A, D and G) Tissues from group I (*n* = 7), exhibiting normal features. (B, E, and H) The polyimide stent in group II (*n* = 7) is still in place, with the tissues around the stent appearing intact. (C, F, and I). The polyimide stent in group III (*n* = 7) is also in place, with the ureteral epithelium enclosing the stent appearing complete. A few inflammatory cells can be seen in the ureteral tissues. The arrows in the images indicate the ureteral stent.

Histopathological analysis of the reconstructive ureteral segments harvested at 12w after surgery showed that the tissue in group I was similar to normal ureteral tissue. In groups II and III, the stents remained in the ureters until the time of tissue sampling. In rats of these two groups, the urothelium, submucosa, and lamina muscularis were complete, while the submucosa and lamina muscularis of the ureters were mildly edematous. No stone formation was found in the reconstructive ureteral segments in groups II and III. Additionally, a few inflammatory cells were present in the ureteral segment tissue (Figure 3).

### Evaluation of kidney weight and renal function

**Figure 4.**
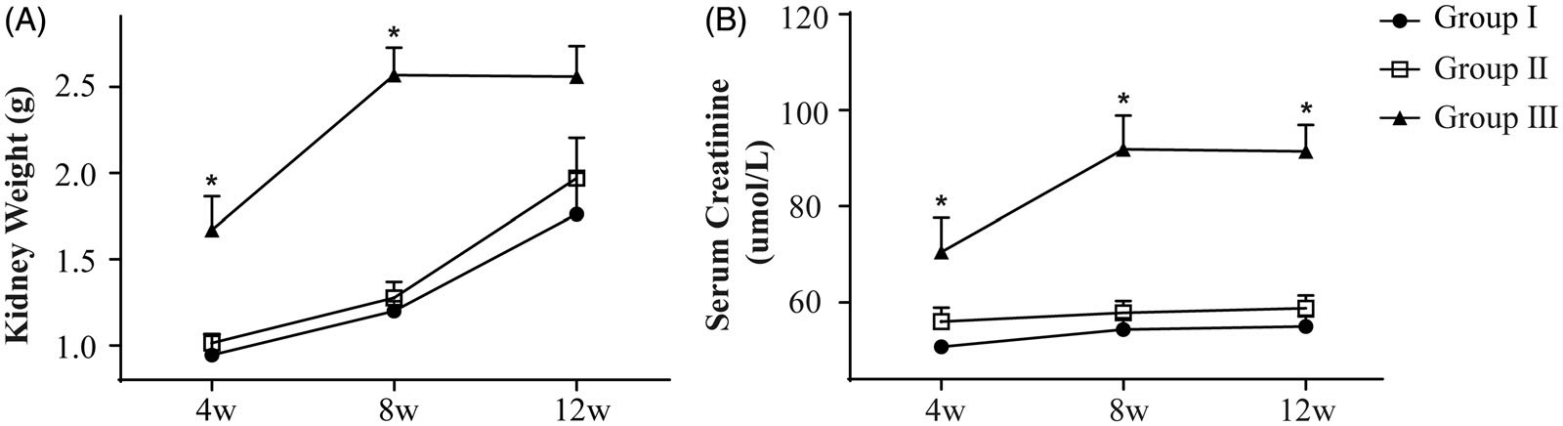
Kidney weight and serum creatinine level. (A) Kidney weight in all three groups increased gradually at postoperative weeks 4, 8, and 12, but it was significantly higher in group III at weeks 4 and 8. (B) The serum creatinine level in group III showed an increasing trend from week 4 to 8, and it was significantly higher than that in the other two groups at postoperative weeks 4, 8, and 12. *p ＜ .05. Each group included 21 animals.

The stent used in this study was beneficial for promoting urethral patency. At postoperative week 4 and 8, the kidney weight of the rats in group III was significantly higher than that in the other two groups (*p* < .05, Figure 4(A)). However, at postoperative week 12, no significant differences in kidney weight were observed among the three groups (*p* > .05, Figure 4(A)). The blood creatinine level of the rats in group III was higher than that in groups I and II at postoperative week 4, 8, and 12 (*p* < .05, Figure 4(B)), but there were no significant differences in the blood creatinine level between groups I and II (*p*>.05, Figure 4(B)).

### Histologic evaluation

**Figure 5.**
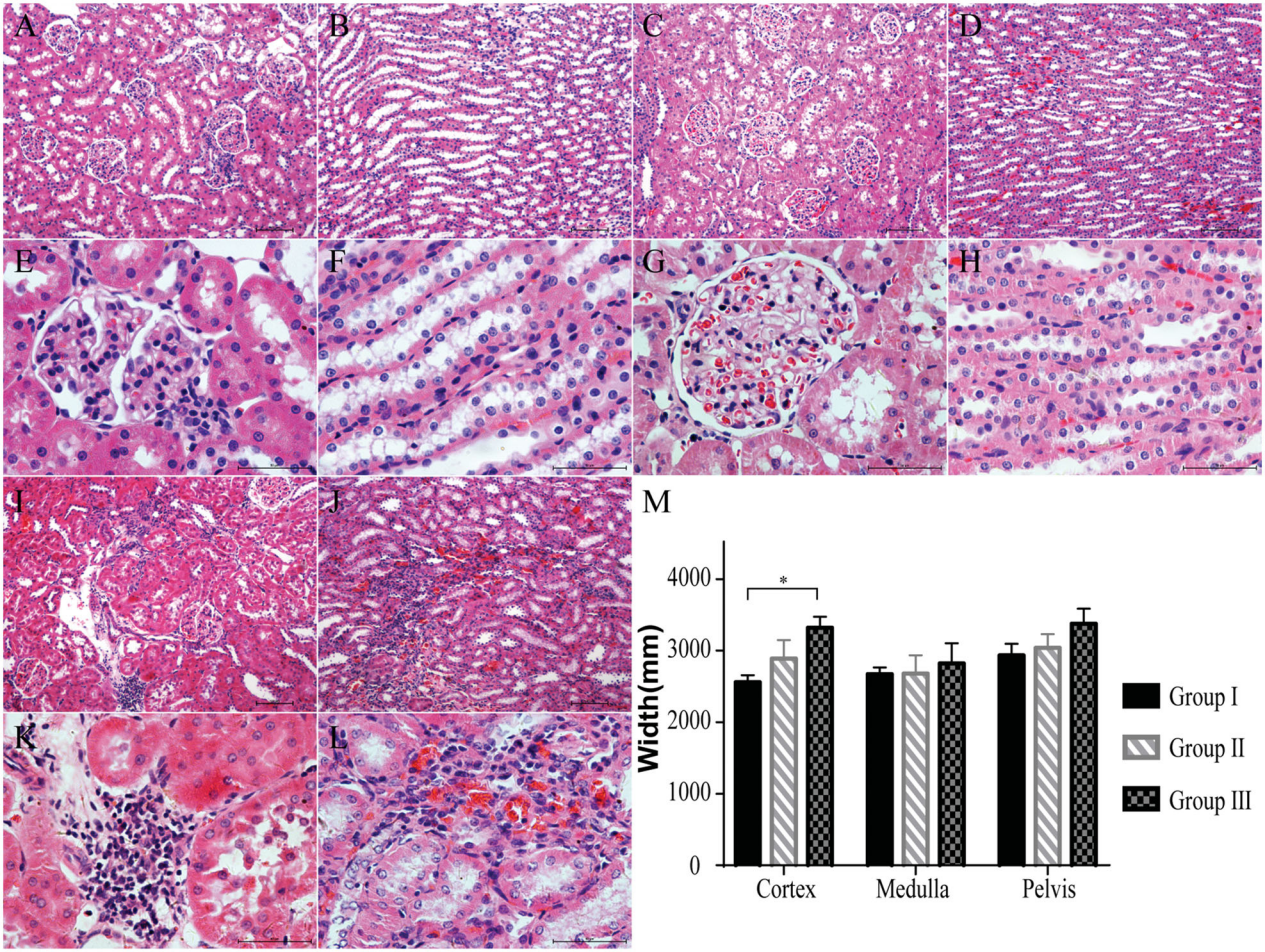
Histologic evaluation of the kidneys at postoperative week 12 (H&E staining). (A, B, 10× and E, F, 40×) Group I (*n* = 7): The renal structure remained intact. (C, D, 10× and G, H, 40×) Group II (*n* = 7): The histological changes in the renal tissues were minimal. (I, J, 10× and K, L, 40×) Group III (*n* = 7): Some of the glomeruli and nephric tubules were destroyed. (M) The width of the cortex was significantly greater in group III than in group I. *p < .05.

Histological examination showed that the kidney tissue features in group I have not changed significantly at postoperative week 12 (Figure 5(A,B,E,F)). In group II, the tubuli and glomeruli were almost completely intact and changes were minimal (Figure 5(C,D,G,H). However, in group III, the number of glomeruli were reduced and infiltrated inflammatory cells were found. Additionally, tubular atrophy and dilatation were observed in the tubuli (Figure 5(I,J,K,L). Finally, the thickness of the renal cortex in group III was significantly higher than that in the other two groups (*p* < .05, Figure 5(M).

## Discussion

In the present study, we used a stent made from polyimide for ureteral anastomosis in a rat model of kidney transplantation. The findings are promising, as the stent was well tolerated and the recipients survived till the end of the experimental period. Histopathological analysis of the reconstructed ureteral segments revealed complete submucosa and lamina muscularis, although a few inflammatory cells were observed. Nonetheless, the histological structure was consistent with that of the normal ureter. But differences were found in the kidney weight among groups. At postoperative week 4 and 8, the kidney weight of the rats in group III was higher than that in the other two groups. Nevertheless, at postoperative week 12, no significant difference in kidney weight was found. And the histological changes in group III were limited. In addition, the blood creatinine level of the rats in group III was higher than that in groups I and II at postoperative week 4, 8, and 12, but no significant differences were found between groups I and II, indicating that the stent insertion would not lead to the rise of blood creatinine level. Hence the differences in kidney weights may attribute to the chronic rejection. In a word, the recovery of renal structure and function is acceptable. Thus, based on these findings, the use of this stent in ureteral reconstruction should be explored further in other animal models as well as in the clinical setting.

The findings of the present study indicate that the polyimide stent had good biocompatibility, as no foreign body reaction or blockages were found in the reconstructed ureter. Accordingly, in our previous report, a cuff made from polyimide had been successfully used in vascular anastomosis without the appearance of a thrombus in the reconstructed vessel [15]. Further, Starr [16] reported that polyimides including chemical sensors and blood pressure sensors are not cytotoxic to human capillary and microvascular endothelial cells. However, an *in vivo* study showed that polyimide-based neural implants produce a slight foreign body response, but have no effect on neural function [17]. Other research has also shown that polyimide intra-fascicular electrodes induced mild scarring and focal chronic inflammation, but are not associated with neurological abnormalities [18]. In the context of ureteral reconstruction, the fastening method with the polyimide stent may also reduce damage to the ureteral wall. Thus, all the findings published so far corroborate the efficacy and safety of using polyimide stents and implants. This is therefore a promising material in the field of biological reconstruction.

When ureteral reconstruction was performed with a teflon stent and fixed with a 5-0 silk suture, stent migration was detected [3]. However, in our experiment, no stent migration was observed. This could be attributed to the passivation of the surfaces of the polyimide stent. Additionally, the 11-0 silk suture tied to the middle of stent and the three silk sutures tied together may also have contributed to the maintenance of the stents in place. These results indicate that the polyimide nature of the stent and the methods used to fix the stent contributed to the survival of the transplanted kidneys and recipients. This technique should therefore be explored in other models and settings.

One limitation to the present study is lack of feasibility verification of polyimide stent using in ureteral anastomosis during kidney allograft transplantation. And a long-term follow-up study is needed.

## Conclusions

Stents made from polyimide are unlikely to damage the ureteral wall and are suitable for ureteral anastomosis during kidney transplantation, as the rats that underwent the procedure showed good survival and tolerance. Further, the modifications made to the polyimide stent (through passivation of its surface) and the changes in the suture fixation method may minimize the possibility of stent migration. Importantly, in the future, these findings could be applied to kidney transplantation procedures in the clinical setting.
